# Central Nervous System Manifestations Associated with Hereditary Transthyretin Amyloidosis: a Narrative Review

**DOI:** 10.1055/s-0046-1817041

**Published:** 2026-03-23

**Authors:** Pedro Ivo Machado Campos de Araujo Costa, Marcelo Maroco Cruzeiro, Arise Garcia de Siqueira Galil, Thiago Cardoso Vale

**Affiliations:** 1 Universidade Federal de Juiz de Fora, Faculdade de Medicina, Programa de Pós-Graduação em Saúde, Juiz de Fora MG, Brazil.; 2 Universidade Federal de Juiz de Fora, Faculdade de Medicina, Departamento de Clínica Médica, Juiz de Fora MG, Brazil.; 3 Universidade Federal de Juiz de Fora, Faculdade de Medicina, Juiz de Fora MG, Brazil.; 4 Universidade Federal de Juiz de Fora, Hospital Universitário, Juiz de Fora MG, Brazil.

**Keywords:** Amyloidosis, Familial, Prealbumin, Cognitive Dysfunction, Central Nervous System

## Abstract

Hereditary transthyretin amyloidosis (ATTRv) is a rare, autosomal dominant, inherited disease caused by variants in the gene encoding the transthyretin (TTR) protein. These variants lead to TTR tetramer destabilization, resulting in the formation and progressive deposition of insoluble amyloid fibrils in various tissues, including those of the central nervous system (CNS). Although previously neglected, the recognition of CNS involvement in ATTRv has become progressively relevant due to prolonged patient survival and the ineffectiveness of the current therapies in addressing CNS synthesis of TTR. The first descriptions of the pathological involvement of the CNS in ATTRv date from the 1960s; however, this topic has not been fully explored. In the present article, the main CNS clinical manifestations of ATTRv, such as transient focal neurological episodes, bleeding complications, leptomeningeal amyloidosis, and cognitive impairment, are reviewed, and the phenotypic variability of this condition is highlighted. A literature review of the PubMed/Medline database was conducted using the following keywords: *hereditary amyloidosis* , *transthyretin amyloidosis* , *familial amyloidosis* , *central nervous system* , *neurological manifestations* , *leptomeningeal amyloidosis* , *cognition* , and *cognitive impairment* . Studies published in the last 15 years, including review articles, prospective observational studies, experimental studies and clinical trials, were evaluated. Improving our understanding of CNS involvement in ATTRv will enable the early identification of neurological symptoms in this condition, which will enhance the understanding of the pathophysiological mechanisms and boost the advancement of research, expanding potential treatments and improving the quality of life of these individuals.

## INTRODUCTION

Hereditary transthyretin amyloidosis (ATTRv) is a rare, autosomal dominant, inherited disease caused by mutations in the gene encoding the transthyretin (TTR) protein, 1 which is synthesized predominantly (approximately 90%) in the liver, but is also produced by the retinal epithelium and secreted into the cerebrospinal fluid (CSF) by the choroid plexus. 2 Functionally, TTR is involved in the transport of the thyroxine hormone and of the retinol binding complex. 3 Although this function is also performed by other serum proteins, such as thyroglobulin and albumin, TTR is the main transporter of vitamin A and thyroxine in the central nervous system (CNS), 4 and it may also play an important role during aging, neuropeptide processing, and neuronal regeneration. 2

Destabilization of the TTR tetramer is the critical step in the pathogenesis of ATTRv, and it is characterized by dissociation of this protein into monomers that fold abnormally and form insoluble amyloid fibrils, which accumulate in the extracellular space of various tissues, particularly the peripheral nerves, heart, and kidneys. 5 The progressive nature of deposition of these fibrils results in various clinical manifestations, such as progressive sensorimotor neuropathy, autonomic dysfunction, heart failure, gastrointestinal dysfunctions, and malnutrition. 3

The CNS is also a target of the disease. The first descriptions of CNS pathological involvement in ATTRv date from the 1960s in Portuguese individuals who were probably carriers of the V30M ( *p.Val50Met* ) variant. Although at the time it was already evident that amyloid could accumulate in large amounts in the leptomeninges of the brain and spinal cord, this finding did not seem to be clinically relevant compared with the myriads of systemic manifestations. 5 Most of the CNS symptoms appear as a late complication, including in patients who underwent liver transplantation and in carriers of the V30M variant. In these latter patients, it takes approximately 14 to 16 years after the onset of peripheral nerve symptoms. 6 7 In patients who are carriers of other variants, severe ocular and CNS phenotypes with little systemic involvement have been reported, termed *oculoleptomeningeal amyloidosis* (OLMA), 5 which can very rarely occur early on. Symptoms of CNS involvement may also include transient focal neurological episodes (TFNEs), known as *amyloid spells* , subarachnoid haemorrhage, hydrocephalus, ataxia, seizures, and cognitive deficits. 2 5 8 These CNS symptoms, except for mild cognitive dysfunction, tend to appear no earlier than 10 years after disease onset in ATTRv patients who are carriers of the V30M variant.

When left untreated, ATTRv has a progressive and fatal course, with a median survival of 10 years. 1 7 The therapeutic options available include liver transplantation, TTR stabilizers (tafamidis and acoramidis) and gene-silencing agents (inotersen, patisiran, vutrisiran, and eplontersen). 9 10 11 These options effectively reduce hepatic TTR production and prolong survival, but do not impact CNS TTR synthesis by the choroid plexus. 1 5 Tafamidis, for example, has low penetration into the CSF, reaching concentrations of only 1.5% of those observed in plasma, whereas inotersen and patisiran do not cross the blood-brain barrier (BBB). 5 The ineffectiveness of RNA silencing in reducing TTR concentration in the CSF has also been demonstrated in an animal model of ATTR (V30M transgenic mice). 12

Given the increased survival of these individuals, the potential impact of CNS manifestations on quality of life, and the limitation of available therapies to effectively address CNS involvement, it is essential to review and consolidate the existing knowledge on the subject. In the current article, we present a narrative review of CNS manifestations of ATTRv, highlighting the gaps in current knowledge and their implications for the clinical practice and future research.

In the present study, the abbreviated nomenclature based on the mature TTR protein sequence was used as the primary form of reference, given its widespread use in the literature, particularly in previous clinical and population-based studies, which facilitates comparison with earlier findings. For clarity, consistency, and adherence to current international standards, the nomenclature recommended by the Human Genome Variation Society (HGVS) is provided in parentheses upon the first mention of each variant.

## METHODS

### Search strategy

A literature search of the PubMed/Medline database was conducted using the following keywords: *hereditary amyloidosis* , *transthyretin amyloidosis* , *familial amyloidosis* , *central nervous system* , *neurological manifestations* , *leptomeningeal amyloidosis* , *cognition* , and *cognitive impairment* . Studies published in the last 15 years that addressed the involvement of the CNS in ATTRv, including review articles, prospective observational studies, experimental studies, and clinical trials, were evaluated.

### Inclusion criteria

The selection process was performed in three stages: initial screening of titles and abstracts, analysis of full texts, and extraction of relevant data for subsequent descriptive analysis. Articles from conference proceedings or symposia were excluded. Only full-texts available in English were selected.

### Data analysis

The first author (PIMCAC) performed the initial screening of titles and abstracts and selected the texts to be read in full. All authors read them and highlighted the information that should be extracted. In-person and remote meetings were held to discuss the highlighted information that should be presented in the current manuscript.

## RESULTS AND DISCUSSION

**Figure 1:**
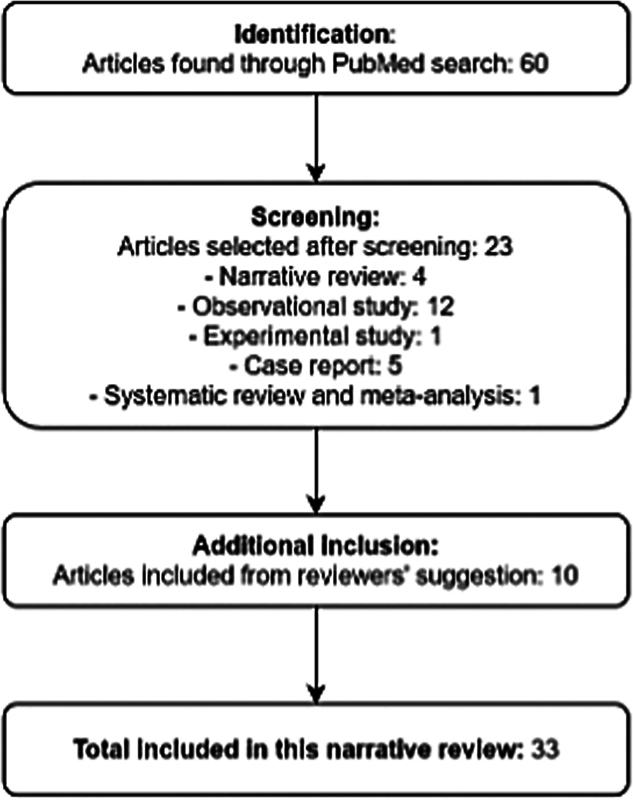
Flow diagram describing the study selection process.

We retrieved 60 articles, and 23 were selected for inclusion in the narrative review ( Figure 1 ). Among the selected studies, observational studies predominated (n = 12), followed by case reports (n = 5), narrative reviews (n = 4), a systematic review with meta-analysis (n = 1), and an experimental study (n = 1). The selected case reports particularly contributed with detailed description of less common clinical presentations and specific phenotypes associated with rare variants. Additionally, ten studies were incorporated based on reviewers' recommendations, due to their relevance to contextualize the topic and characterize the clinical manifestations across different phenotypes.

For a clearer demonstration of the results, they are presented according to their frequency in the articles included in the present review. Table 1 provides a summary of the main clinical manifestations of CNS involvement in ATTRv and the possible pathophysiological mechanisms, which will be further detailed in the subsequent sections of the current review.

**Table 1:** Central nervous system manifestations in ATTRv: summary of clinical features and pathophysiological mechanisms

Clinical syndromes	Possible pathophysiological mechanisms	Frequency and clinical features	Additional information
Transient focal neurological episodes	Focal epileptic seizures or cortical spreading depression triggered by hemoglobin degradation products in the subarachnoid space and superficial cortex, with additional proposed mechanisms including ischemia and vasospasm. 13 14	Variable prevalence in posttransplant V30M: 31%, 6 20.8%, 7 and 11.3%. 13	Transient episodes, negative (weakness, dysarthria, sensory disturbances) or positive (involuntary movements, paresthesia, scotomas). 5 6 13 Mimic TIAs but evolve more slowly, lasting minutes to hours. Associated with TTR-CAA findings on neuroimaging. 7
Hemorrhagic complications	TTR-related CAA causing vascular fragility and lobar hemorrhage. 6 15	8.3% of 48 transplanted patients with V30M 7 (3 fatal, 1 microhemorrhage); 1,15% (1 case) in 87 transplanted patients. 6	Intracerebral hemorrhages related to TTR deposition in a pattern similar to Aβ-CAA. 6 15 16 17
Leptomeningeal amyloidosis	Amyloid deposition in leptomeninges and CNS vessels. 13 18	Rare; manifestations include headache, seizures, cognitive impairment, stroke, pyramidal signs, and ocular symptoms (vitreous opacities, glaucoma, retinal angiopathy). 5 18 19 20 21	Associated with destabilizing variants: V30G, L12P, F64S, A36P, G53E, Y69H, A25T, Y114C, D18G, 2 22 and others. Course may be rapid or insidious.
Cognitive impairment	Amyloid angiopathy-related inflammation, direct neuronal toxicity, and oxidative stress. 7 Mesial temporal and posterior atrophy. 2 23 Reduced cortical thickness in the orbitofrontal, temporal, parietal, and insular regions. 24	19.7% of the patients (573 evaluated, with a mean age of 46.5 years); 25 in transplanted V30M: episodic memory – 31%; executive functions – 25%; and attention – 19%. 1	May appear early, even in asymptomatic carriers, and not directly correlated with disease duration or severity. 24 26
Cranial nerve and brainstem dysfunction	Direct amyloid deposition in motor and sensory cranial and auditory pathways. 27 28 29 Additional mechanisms involving cochlear structures and cortical hyperexcitability underlying phenomena such as palinacousis. 30	Dysphagia – 17%; oropharyngeal symptoms – 40%; and dysphonia – 36%, reported in mixed cohort including patients with ATTRv, ATTRwt and AL; 27 hearing loss in 89% of ATTRv patients. 28	Involvement of cranial nerves V, VII, IX, X, XI. 27 28 29 30 Hearing loss common, possibly due to deposits in the vestibulocochlear nerve. 28 Palinacousis reported in a posttransplant patient. 30

Abbreviations: AL, light-chain amyloidosis; ATTRv, hereditary transthyretin amyloidosis; ATTRwt, wild-type transthyretin amyloidosis; Aβ, amyloid Beta; CAA, cerebral amyloid angiopathy; CNS, central nervous system; TIA, transient ischemic attack; TTR, transthyretin.

Note: Variants are presented using an abbreviated nomenclature based on the one-letter amino acid code and the residue numbering corresponding to the mature transthyretin protein. The following correspondences apply: V30M ( *p.Val50Met* ), V30G ( *p.Val50Gly* ), L12P ( *p.Leu32Pro* ), F64S ( *p.Phe84Ser* ), A36P ( *p.Ala56Pro* ), G53E ( *p.Gly73Glu* ), Y69H ( *p.Tyr89His* ), A25T ( *p.Ala45Thr* ), Y114C ( *p.Tyr134Cys* ), and D18G ( *p.Asp38Gly* ).

### Transient focal neurological episodes

The most reported symptoms of CNS involvement in ATTRv due to the V30M variant were TFNEs, 6 which refer to stereotyped symptoms of short duration and, in most cases, a negative nature, characterized by temporary loss of function lasting several minutes, such as weakness, sensory disturbance, and dysarthria. 5 13 However, positive symptoms may also be present, and they include involuntary limb movements, paresthesia and scintillating scotomas. 6 13

Studies have shown a variable prevalence of TFNEs in patients with V30M variants after liver transplantation, with values of 31% (27/87), 6 20.8% (10/48), 7 and 11.3% (6/53) 13 being reported.

**Figure 2:**
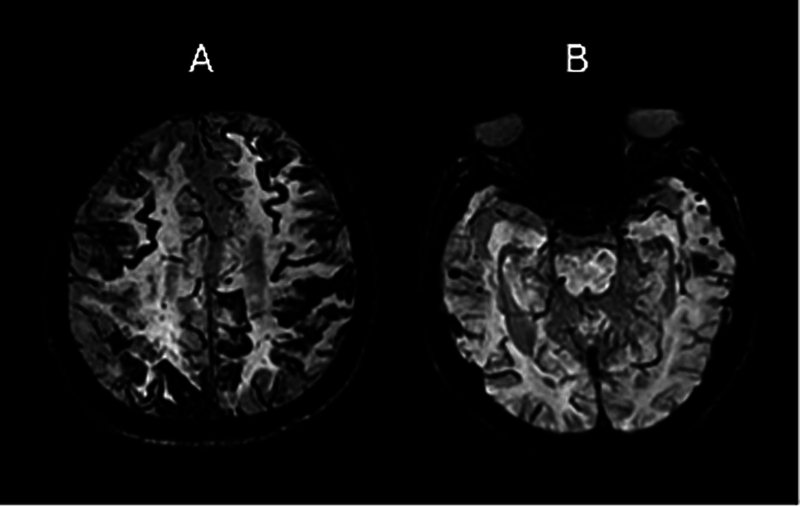
Cortical superficial siderosis ( **A** ) and lobar cerebral microbleeds ( **B** ) on susceptibility-weighted imaging (SWI), in the context ofβBeta-amyloid-related cerebral amyloid angiopathy. **Note:** Adapted from part of Figure 1 in Schwarz et al. 31

Transient focal neurological episodes can mimic transient ischemic attacks (TIAs), but they present clinical and neuroimaging features that help their differentiation. While TIAs have a sudden onset and generally rapid resolution, the progression of TFNE symptoms occurs over minutes, lasting from minutes to a few hours. Transient focal neurological episodes often involve migratory symptoms, commonly associated with neuroimaging findings characteristic of amyloid angiopathy ( Figure 2 ), such as sulcal subarachnoid hemorrhage and siderosis. 7

A comparative study 14 of the clinical characteristics of TFNEs in patients with cerebral amyloid angiopathy (CAA) associated with TTR and amyloid Beta (Aβ) revealed that, although speech disorders and paresthesia were frequent in both groups, visual symptoms were present in four of the seven individuals in the CAA group, whereas no patient in the Aβ group presented such manifestation, suggesting a possible relationship with the predominance of amyloid deposits in the occipital region in the CAA group. Corroborating these findings, a study 32 involving a large cohort of 248 individuals with Aβ-associated CAA only identified visual symptoms in 7.7% of the patients, a frequency lower than that observed for sensory (74.2% of the patients) and motor symptoms (43.5% of the patients), although 1 patient in whom visual symptoms occurred in isolation was described, highlighting the clinical heterogeneity of the TFNEs. These findings reinforce the importance of additional investigation into the anatomical distribution of amyloid deposits and their clinical correlation with different forms of CAA through the use of advanced neuroimaging techniques, such as positron-emission tomography with amyloid markers (amyloid-PET). 14

Hypotheses for the pathophysiological mechanisms of these clinical events include ischemia, vasospasm, epilepsy, and cortical spreading depression. 14 In a case series of 53 patients with postliver transplantation ATTRv, the most likely reported pathogenic mechanisms were focal epileptic seizures or cortical spreading depression, both resulting from focal accumulation of hemoglobin degradation products in the subarachnoid space and in the superficial layers of the cortex. 13

The occurrence of TFNEs is closely related to disease duration. 6 In liver-transplantation patients with the V30M variant, symptoms appeared, on average, 14.6 6 to 16.6 years 7 after disease onset, a period longer than the life expectancy of nontransplant patients (10.9 years; 95%CI: 10.5–11.3). However, evidence has already demonstrated early deposits of TTR in the CNS approximately 3 years after disease onset, progressing along the meninges to the meningocortical vessels and, later, to the superficial cortical parenchyma. 6 In these individuals, prolonged survival after liver transplantation seems to provide time for greater deposition of TTR in the CNS, resulting in the previously-described neurological symptoms. 7 33

Although TFNEs represent the most common CNS manifestation of ATTRv, and their early recognition can prevent more severe intracranial hemorrhages, the literature on this topic remains limited due to the scarce number of patients described. 7 Understanding these events has relevant therapeutic implications, raising concerns regarding the use of aspirin or anticoagulants due to the increased risk of bleeding complications. 6 7

### Bleeding complications

Bleeding complications have been reported in studies involving individuals with ATTRv undergoing liver transplantation, and these complications represent a significant outcome in this patient population. In a cohort of 48 transplanted patients, Dardiotis et al. 7 (2020) reported CNS manifestations in 10 patients, 4 (8.3%) of whom developed hemorrhagic complications: 3 with fatal cerebral hemorrhages and 1 with subclinical microhemorrhages on magnetic resonance imaging (MRI) scans. Only one patient presented TFNEs before the bleeding event. Similarly, in a cohort of 87 transplanted patients with the V30M variant, Maia et al. 6 (2015) reported CNS manifestations in 27 individuals, among whom 5 experienced stroke. Of these, 1 patient (1.15%) presented with a right frontal cerebral hemorrhage, confirmed histopathologically as CAA associated with TTR deposition. No relevant abnormalities were observed on cranial computed tomography (CT) scans in the remaining four stroke cases. However, the presence of minor bleeding events or microhemorrhages could not be excluded, as MRI was contraindicated due to pacemaker implantation in all patients. 6

Impairment of the integrity of cerebral vessels, especially in lobar regions, has been previously described in CAA because of Aβ accumulation. 16 Recognized by the deposition of amyloid protein in the middle and adventitia layers of small- and medium-sized arteries, as well as arterioles, CAA may also affect the leptomeningeal cortical veins. 17 The pattern of TTR deposition in the CNS, which begins in the meninges and progresses to the meningocortical vessels and superficial parenchyma, is remarkably similar to that of CAA associated with Aβ accumulation, indicating a possible shared underlying mechanism in the predisposition to cerebral hemorrhage. 6

In a neuropathological study involving 16 autopsied brains from individuals with ATTRv (of the V30M variant), Taipa et al. 15 (2023) demonstrated that TTR deposition in the CNS follows a defined topographical progression. The samples underwent conventional histological staining (hematoxylin-eosin, Perls' Prussian blue, and Congo red) and immunohistochemistry with antibodies against TTR, Aβ, Tau, TDP-43: TAR DNA-binding protein 43, and Alpha-synuclein, enabling the characterization and mapping of amyloid deposits. Deposition was initially observed in the leptomeninges and subarachnoid vessels, subsequently extending to perforating cortical vessels and subpial regions, and, at more advanced stages, to subependymal and basal ganglia vessels. Early and marked involvement of the brainstem and spinal cord was also noted, even in the initial stages of the disease. Despite extensive superficial deposition, no amyloid deposits were identified in the deep parenchyma, nor were significant vascular lesions or superficial cortical siderosis observed, suggesting that although the distribution pattern resembles that of Aβ-related CAA, the vascular consequences may differ in frequency and severity. 15

In clinical trials evaluating disease-modifying therapies for ATTRv, CNS hemorrhagic complications have been infrequently reported, and no consistent increase in risk has been demonstrated. In studies with tafamidis, 34 no intracranial hemorrhages were documented, and the safety profile remained favorable throughout the long-term follow-up. In the pivotal Efficacy and Safety of Inotersen in Familial Amyloid Polyneuropathy (NEURO-TTR) trial, 35 36 inotersen was associated with rare but severe intracranial hemorrhages, including 1 fatal case related to thrombocytopenia, with platelet count < 10,000/mm ^3^ , prior to the implementation of weekly platelet monitoring; after this measure was adopted, no further severe thrombocytopenia-related hemorrhages occurred, and the overall bleeding incidence was comparable to that of placebo. In the eplontersen trial, 37 a fatal intracerebral hemorrhage occurred after ten doses in a patient with normal platelet count and coagulation parameters, deemed consistent with the known natural history of ATTRv and possibly related to underlying CAA. Conversely, in APOLLO: The Study of an Investigational Drug, Patisiran (ALN-TTR02), for the Treatment of Transthyretin (TTR)-Mediated Amyloidosis, 38 patisiran was not associated with hemorrhagic complications or platelet abnormalities, and adverse events were mainly mild-to-moderate infusion reactions and peripheral edema, supporting a favorable hematological safety profile. Similarly, the HELIOS-A: A Study of Vutrisiran (ALN-TTRSC02) in Patients With Hereditary Transthyretin Amyloidosis (hATTR Amyloidosis), which evaluated the efficacy and safety of vutrisiran, did not report CNS hemorrhagic events. 39 Collectively, these findings suggest that while ATTRv-related CAA may predispose to intracerebral hemorrhage, particularly in the setting of drug-induced thrombocytopenia, the overall risk in clinical trial populations appears low, highlighting the importance of vigilant hematological monitoring, particularly for patients treated with inotersen.

### Leptomeningeal amyloidosis

Highly-destabilizing variants in the *TTR* gene favor deposition of amyloid in the CNS, culminating in a currently-untreatable clinical presentation, *leptomeningeal amyloidosis* (LA). 18 The following mutations are associated with leptomeningeal involvement: V30G ( *p.Val50Gly* ), L12P ( *p.Leu32Pro* ), F64S ( *p.Phe84Ser* ), A36P ( *p.Ala56Pro* ), G53E ( *p.Gly73Glu* ), Y69H ( *p.Tyr89His* ), 2 A25T ( *p.Ala45Thr* ), Y114C ( *p.Tyr134Cys* ), D18G ( *p.Asp38Gly* ), 22 I107M ( *p.Ile127Met* ), 8 and L55R ( *p.Leu75Arg)* . 40 The leptomeningeal presentation of ATTRv is a severe form of the disease, and its diagnosis may be delayed in nonendemic regions. 41

In the spectrum of LA, a rare phenotype characterized by the predominance of ocular symptoms associated with CNS involvement has been described as OLMA. 5 18 19 20 This condition was explored by Goren et al. 21 (1980), who investigated a family of German descent residing in Ohio (United States), in which symptoms of ocular and CNS dysfunctions predominated. At the time of publication, in 1980, only 2 other families with similar symptoms had been studied.

The clinical presentation of OLMA is heterogeneous and may include ocular dysfunction with visual deterioration, often associated with vitreous opacities, and CNS disorders such as stroke, cognitive impairment, epileptic seizures, pyramidal and cerebellar signs, hearing loss, headache, central apnea, and visual and speech disorders. 5 21 The symptoms predominantly begin in the third to fifth decades of life, and they may progress rapidly and lead to death within months, or they may progress insidiously with a more protracted course of up to 3 decades. 5

Quintanilha et al. 42 (2020) reported the first Brazilian case of OLMA associated with the Y69H variant, a rare mutation in the *TTR* gene. The patient, a 37-year-old man, presented with recurrent transient neurological episodes, including right-sided paresthesia and weakness, headache, mental confusion, and aphasia, in addition to a single isolated epileptic seizure. Brain and spinal MRI scans revealed diffuse leptomeningeal enhancement, and a meningeal biopsy confirmed amyloid deposits. Despite the absence of ophthalmologic symptoms at disease onset, increased latency was identified on visual evoked potentials. There was no evidence of peripheral or cardiac involvement, reinforcing the CNS-restricted phenotype observed with this specific variant. 42

Although ophthalmological manifestations in ATTRv are frequently associated with leptomeningeal involvement, they may also occur in isolation or precede other neurological symptoms. Variants such as V30M, V30G, F64S, and Y114C are linked to characteristic ocular abnormalities, including vitreous opacities, open-angle glaucoma, pupillary changes, and retinal angiopathy, reinforcing the association between ocular findings and neurological involvement in ATTRv. 43

Individuals with OLMA may also present with significant and progressive cognitive impairment, progressing to dementia, episodes of mental confusion, and irritability. A neuropathological analysis 21 revealed severe diffuse neuronal loss, particularly in the superficial layers of the cerebral cortex; diffuse subpial degeneration and gliosis affecting the white matter, and extensive amyloid deposits in the leptomeninges and subarachnoid vessels.

Several case series and specific reports reinforce the clinical heterogeneity of OLMA, with new publications pointing to unprecedented associations of specific variants in the *TTR* gene with this form of presentation. An example is the I107M variant, for which the association with the OLMA phenotype was first described in 2018, 8 and the L55R variant, described 40 in 2020 in two siblings, whose presentation included marked visual loss, significant weight loss, and severe autonomic neuropathy, evidencing the multiplicity of manifestations.

### Cognitive involvement

An analysis of the publications on the cognitive performance of individuals with ATTRv reveals a comprehensive picture of deficits, with predominant impairment in executive functions, attention, and memory. The relative preservation of some functions and the variability between different subgroups of patients indicate that the cognitive profile of ATTRv is complex and multifaceted, and further studies are needed for a better understanding. 1 2 5 7 26

Episodic memory was the most affected cognitive domain (31%), followed by impairment in executive functions (25%) and attention (19%), with preservation of visuoconstructive functions, in liver-transplanted V30M carriers who underwent neuropsychological assessment. 1 The patients were evaluated through the California Verbal Learning Test, Trail Making Test (TMT), Toulouse-Pieron Test of Concentrated Attention, Clock Drawing Test, and Mini-Mental State Examination (MMSE). 1 The inattention index was low in many patients, demonstrating the involvement of frontal neuronal networks in ATTRv patients. The MMSE score was relatively high, probably reflecting the low sensitivity of the test in the evaluation of executive dysfunction; however, the MMSE score may also have been influenced by a ceiling effect, considering the relatively young age and level of schooling of the participants, masking subtle deficits that were only detected by other more sensitive neuropsychological assessments. 1

In 2018, the first systematic neuropsychological evaluation of patients with untreated V30M variant was published. 26 In this study, which evaluated 340 V30M variant carriers (180 symptomatic and 160 asymptomatic), the cognitive assessments included the Dementia Rating Scale 2 (DRS-2), an auditory verbal learning test, semantic and phonemic fluency tests, TMT, and screening for depression and anxiety with a self-reported questionnaire. Cognitive dysfunction was more frequent among symptomatic and older patients, with verbal learning, memory, and executive functions being the most affected domains. The study demonstrated a particular vulnerability of executive and memory dysfunction in older symptomatic untreated patients, with approximately half with deficits in at least one or both domains. Among younger subjects (younger than 50 years), a statistically significant difference was not observed between symptomatic and asymptomatic patients. In asymptomatic carriers of the V30M variant, a greater risk of cognitive dysfunction was not observed, regardless of age. 26

Durmuş et al. 2 (2021) assessed the cognitive functions of ATTRv patients followed between 1995 and 2020 and observed that cognitive impairment was common, representing a significant component of the broad clinical spectrum of the disease. In this Turkish study, the neuropsychological profile indicated that patients exhibited deficits in at least one cognitive domain, with long-term verbal memory and long-term visual memory being the most affected, in addition to executive and visuospatial functions, except for one patient who presented with isolated carpal tunnel syndrome. 2

Torres et al. 44 (2021), in a Brazilian study conducted with patients from the state of Ceará, assessed the cognitive functions of individuals carrying the I107V ( *p.Ile127Val* ; 6 participants) and Y60A ( *p.Thr80Ala* ; 2 participants) variants, with a median age of 30 years, comparing them to a control group of 16 individuals. Although lower performance was observed in the domains of verbal fluency, memory, and language, no statistically significant differences were found. The incidental memory impairment identified through the Brief Cognitive Screening Battery may reflect attentional dysfunction, a domain frequently affected in previous studies, according to the authors. 44

More recently, another Brazilian study 24 evaluating 29 symptomatic individuals with ATTRv, 36.4% carrying the V30M variant and 27.6%, the V122I ( *p.Val142Ile* ) variant, compared to 26 controls, identified cognitive impairment characterized by lower performance in executive functions, verbal and visual memory, visuospatial abilities, language, and the MMSE. The individuals had a mean disease duration of 6.7 ± 7.1 years, with no correlation observed involving cognitive performance and either disease duration or disability level, suggesting that cognitive alterations may emerge early and regardlessof clinical progression. 24

In a systematic review conducted by Senem et al. 25 (2025), among the 573 patients evaluated across studies, 19.7% presented with cognitive dysfunction, with a mean age of 46.5 ± 10.1 years. The most frequently affected domains were memory, attention, processing speed, and executive functions, with additional reports of visuospatial deficits, language impairment, and reduced IQ in rarer variants. Cognitive dysfunction was observed not only in advanced cases of the V30M variant, including liver-transplant recipients, but also in asymptomatic carriers and individuals with less common mutations, suggesting a broader spectrum of CNS involvement than previously recognized. 25

### Cranial nerve and brainstem disorders

Symptoms involving the cranial nerves and brainstem are less described than those previously mentioned. A prospective observational study 27 involving 95 patients with a cardiac phenotype, including 19 individuals with ATTRv, 40 with wild-type transthyretin amyloidosis (ATTRwt), and 36 with light-chain amyloidosis (AL), revealed dysphagia (prevalence of 17%), oropharyngeal symptoms (40%) and dysphonia (36%), suggesting involvement not only in the tissues directly involved in the phenotype but also in the motor and sensory cranial nerves related to swallowing and phonation (nerves V, VII, IX and X). 27 The same authors 28 also reported auditory impairment in 89% of the 19 individuals with ATTRv evaluated; the mechanisms underlying auditory impairment may involve deposition of amyloid in the vestibulocochlear nerve and in the CNS, as well as in the cochlear hair cells and structures of the middle ear.

A peculiar auditory involvement, consisting of echo or repetition of the last sound heard, was recently described in a patient with ATTRv who underwent liver transplantation; this condition occurred approximately 48 hours after the onset of a TFNE characterized by motor aphasia and hemiparesis. 30 The condition, known as *palinacousis* , is characterized by the paroxysmal maintenance of a sound stimulus after its termination, and it may be associated with cortical dysfunctions, notably in the left temporal lobe; palinacousis involves mechanisms of hyperexcitability of the auditory cortex, although the pathophysiology is still poorly understood. 30

Additional evidence regarding the vulnerability of cranial nerves in ATTRv was reported in a Brazilian observational series investigating the I107V variant. 29 Batista et al. 29 (2022) systematically evaluated 14 symptomatic patients and observed clinical manifestations resulting from the involvement of bulbar cranial nerves in all studied individuals, notably dysphagia, dysphonia, dysgeusia, and tongue atrophy. Two patients progressed to require gastrostomy and one required tracheostomy. Most patients evaluated also demonstrated involvement of cranial nerves V, VII, and XI, confirmed by electrophysiological studies. Although histopathological analyses were not performed, the authors 29 suggest that these clinical manifestations likely result from amyloid deposits directly in the cranial nerves rather than from local deposits, as no macroscopic abnormalities were observed upon laryngoscopic inspection, and symptoms were not restricted to the larynx.

### Contribution of neuroimaging

The advancement of neuroimaging techniques has aided in understanding the implications of amyloid deposition in the neurological manifestations of ATTRv, stimulating investigation into the pathophysiological mechanisms underlying the cognitive impairment that occurs in this disorder. 2 Long-term memory deficits are associated with mesial temporal atrophy and visuospatial deficits are associated with posterior atrophy. 2 The presence of cortical atrophy, associated with other structural changes, suggests that amyloid deposition in CNS may cause long-term neurological dysfunction, reinforcing the need for continuous monitoring of these individuals. 23

**Figure 3:**
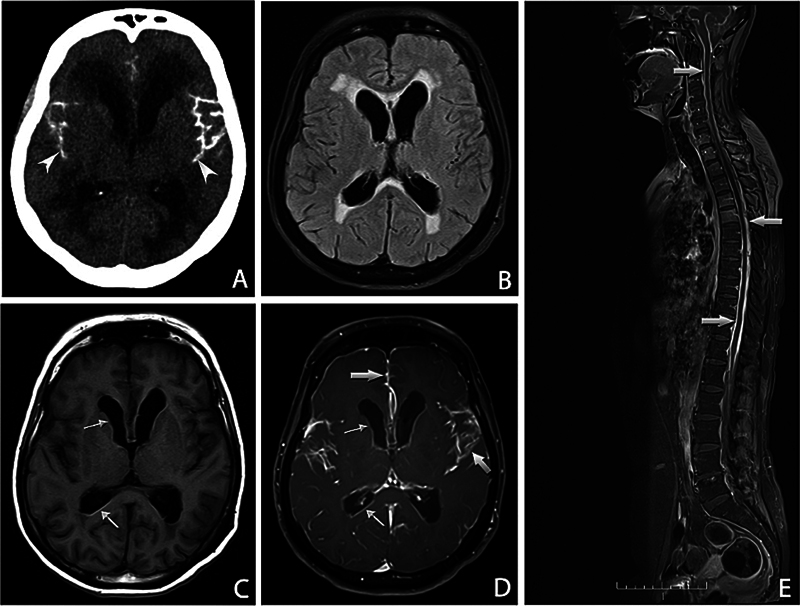
Note: reproduced from Muthukumarasamy et al. 41 Neuroimaging findings: axial computed tomography (CT) scan ( **A** ) showing sulcal calcifications (arrowheads) and obstructive hydrocephalus upon the initial presentation. Images **B–F** are from a subsequent brain and whole spine magnetic resonance imaging (MRI) scan. Fluid-attenuated inversion recovery (FLAIR) sequence ( **B** ) demonstrating ventricular dilatation with associated periventricular hyperintensity. The thin arrows highlight ependymal hyperintensity on the precontrast T1-weighted image ( **C** ), with corresponding enhancement on the gadolinium-enhanced T1-weighted sequence ( **D** ). Thick arrows in the contrast-enhanced brain ( **E** ) and spine ( **F** ) images demonstrating extensive leptomeningeal enhancement.

The most consistent finding of LA associated with most genotypes is leptomeningeal enhancement ( Figure 3 ), and in patients who are carriers of some variants, superficial cortical siderosis – A25T, A36P, D18G, A45T ( *p.Ala65Thr* ) and G53E ( *p.Gly73Glu* ) – and hydrocephalus – L12P, V30G, D18G and G53R ( *p.Gly73Arg* ) – may also be found. 41

The leptomeningeal enhancement identified via conventional MRI methods is not specific to detect amyloid deposits, but PET with Pittsburgh Compound B (11C-PiB-PET) is a potentially-useful tool to detect CAA. 33 A study with 15 patients with ATTRv after liver transplantation, 13 including 5 with CNS symptoms, revealed increased retention of the radiopharmaceutical in the brain of 11 individuals, with greater retention in those with longer disease duration and predominant retention in leptomeningeal vessels, leptomeninges, the occipital cortex and cerebellum, without significant involvement of the brain parenchyma, a distinct accumulation pattern compared with Alzheimer's disease (AD), in which there is strong accumulation in the frontal, parietal, and temporal cortices, the posterior cingulate gyrus, and the precuneus. In this study, 13 there was no direct correlation between the presence of amyloid deposits and lesions detectable by conventional MRI, suggesting that 11C-PiB-PET is a more sensitive tool for the evaluation of CAA and ATTR.

Due to the short half-life of 11C-PiB, the use of flutemetamol (18F) may yield advantages due to its longer half-life, enabling greater flexibility in neuroimaging protocols, and according to previous experiences in the detection in the detection of AB protein deposition Aβ deposition in patients with AD, facilitating comparisons with the deposition patterns in ATTRv. 33 A distinct pattern of predominant cerebellar involvement is observed in 60% of the individuals, with an inverse correlation with age at disease onset, in contrast to the typical pattern found in AD, in which diffuse cortical deposition predominates. 33

Through quantitative neuroimaging using 3-Tesla brain MRI, Senem et al. 24 (2025) investigated the relationship between cortical morphology and cognition in individuals with ATTRv. Despite the small sample size and the short disease duration among the participants, this study is remarkable for its pioneering approach. Reduced cortical thickness in regions such as the lateral orbitofrontal cortex, middle temporal gyrus, supramarginal gyrus, insula, pars opercularis, and global mean cortical thickness was associated with poorer executive performance, as assessed by the TMT-B. In the visuospatial domain, performance on the Rey-Osterrieth Complex Figure Test (ROCF) showed a positive association with the integrity of the lingual gyrus and cuneus cortex. Language, measured by the vocabulary subtest, was related to the thickness of the precentral gyrus and to white matter integrity, as indicated by increased mean diffusivity in the internal and external capsules. Verbal memory, assessed by the Rey Auditory Verbal Learning Test (RAVLT), correlated positively with multiple cortical regions, including the inferior temporal gyrus, pars opercularis, insula, lingual gyrus, fusiform gyrus, lateral orbitofrontal cortex, and postcentral gyrus. These findings reinforce the presence of structural brain impairment associated with specific cognitive deficits, providing valuable insights into CNS involvement in ATTRv.

In conclusion, it is important to understand the spectrum of clinical presentations of ATTRv, which includes phenotypes with neuropathic, cardiac or CNS predominance. Cognitive impairment and other CNS manifestations in ATTRv typically arise in two main clinical scenarios: as a late complication in classic neuropathic phenotypes—particularly among V30M carriers with prolonged survival after liver transplantation—or as early features in oculoleptomeningeal phenotypes associated with highly-destabilizing variants. These findings underscore the clinical complexity and phenotypic diversity of the disease, highlighting the need for focused attention on the underlying pathophysiological mechanisms and the limitations of current therapies. Although liver transplantation was the first therapeutic approach to treat ATTRv, enabling prolonged survival of the patients, it does not prevent the progression of CNS TTR deposition, as reflected by greater CNS involvement in those with longer disease duration. However, investigations dedicated to CNS involvement in individuals undergoing treatment with disease-modifying therapies, such as TTR stabilizers and gene silencers, are lacking. The clinical impact of these therapies on CNS symptoms is still uncertain. More studies are needed to broaden the understanding of the mechanisms that lead to CNS involvement, which would contribute to the identification of therapeutic targets and the development of target drugs with the ability to overcome the BBB to more effectively address the full spectrum.
